# National identity as a lens on social inequality: a cross-national analysis of support for native employment priority

**DOI:** 10.3389/fsoc.2025.1657087

**Published:** 2026-02-16

**Authors:** Simona Guglielmi

**Affiliations:** Department of Social and Political Sciences, University of Milan, Milan, Italy

**Keywords:** EVS, exclusionary attitudes, labour market inequality, national identity, nativism, perceived immigration threat, chauvinism, structural equation modelling

## Abstract

**Introduction:**

Public support for restricting immigrants’ access to welfare and employment—often described as welfare or labour market chauvinism—has become a salient political issue across Europe. This article investigates how symbolic boundaries of national identity shape these exclusionary preferences, focusing on the belief that native citizens should be prioritised over immigrants when jobs are scarce.

**Methods:**

Inspired by the Verkuyten’s *Group Identity Lens* model (2009, 2018), it tests a structural equation model, which links ethnic and civic conceptions of nationhood to support for native employment priority through perceived economic and cultural threats and intergroup distrust. Using data from the 2017 European Values Study and structural equation modelling across seven European countries (France, Germany, Great Britain, Hungary, Italy, Poland, and Portugal), the study tests two core hypotheses: the *nativist spiral*, whereby ethnic-majoritarian identity fuels exclusion via threat and distrust; and *two-faced civility*, which suggests civic identity can reduce distrust but also heighten threat perceptions.

**Results:**

Results confirm the strong and consistent role of ethnic identity in driving exclusionary attitudes. Civic identity shows more ambivalent effects, varying by national context.

**Discussion:**

These findings highlight how both forms of national identity can act as symbolic filters through which inequalities are justified and solidarity is selectively applied.

## 1 Introduction

The increase in immigration to Europe over the past two decades has intensified debates over welfare sustainability, posing significant challenges to the inclusiveness of European welfare states (Harris and Römer, 2023; Saar et al., 2022; Harell et al., 2021). Although the rights of European Union citizens have been harmonised across member states, national governments retain greater authority over the welfare entitlements of third-country nationals (Kramer and Heindlmaier, 2021). These policy divergences have contributed to the emergence of a dualised welfare state that provides unequal protection to citizens and non-naturalised immigrants. When combined with labour market dualism—i.e., the division between secure, well-paid jobs predominantly accessible to natives and precarious, low-paid jobs often filled by immigrants—this increases the risk of poverty and marginalisation among immigrant populations. However, these disparities are often masked/misperceived by public narratives that frame immigrants as job competitors or attribute their disadvantaged position to low qualifications or insufficient effort, rather than systemic discrimination (May and Czymara, 2024; Leruth et al., 2024).

Such material inequalities are increasingly intertwined with cultural and symbolic dynamics. Across Europe, public discourse often reframes these issues through the prism of national belonging, casting cultural diversity—especially when linked to international migration—as problematic (Gattinara and Morales, 2017; Antonsich and Petrillo, 2019). Within this context, ethnocentric and monocultural understandings of national identity are regaining prominence (Kaufmann, 2019; Rodi et al., 2023), following a period in which supranational integration and multicultural paradigms predominated. In the decades after the Second World War, processes of European integration and globalisation fostered cosmopolitan ideals of postnational citizenship (Habermas, 1992, 2001), while multicultural frameworks sought to accommodate internal cultural diversity within liberal democracies (Kymlicka, 1995; 2024). The emergence of this “new nationalism” has become a defining hallmark of contemporary politics, especially since the 2008 financial crisis and the 2015 refugee crisis (Mudde and Rovira Kaltwasser, 2018; Betz, 2022). At its core lies a discourse that legitimises preferential treatment for native citizens, often encapsulated in slogans such as “co-nationals first” (Halikiopoulou and Vlandas, 2022; Rathgeb and Busemeyer, 2022). A prominent manifestation of this discourse is welfare chauvinism (or welfare nationalism): the belief that social welfare benefits should be reserved for (native) citizens, while excluding immigrants. In the literature (see Careja and Harris, 2022, for a review), the term is used both to characterise policies that institutionalise such preferences—for example, by linking welfare access to citizenship or long-term residency—and to describe exclusionary public attitudes, often mobilised by national-populist radical right parties (Hutter and Kriesi, 2022). Similarly, some scholars used “labour market chauvinism” to describe support for the idea that co-nationals should have priority in accessing available jobs (Ferrera and Pellegata, 2018; Kuhn and Kamm, 2019).

Both welfare and labour market chauvinism are frequently framed as a reaction by ethnic majorities who feel their cultural or social dominance is under pressure (Betz, 2022; Kaufmann, 2019; Brubaker, 2020; Hutter and Kriesi, 2022). Alternative terms, such as welfare restrictiveness (Degen et al., 2019), exclusive solidarity (Lefkofridi and Michel, 2017), or selective solidarity (Koning, 2013; Magni, 2021), have also been employed. These formulations tend to emphasise material inequalities in welfare/labour market access, often neglecting the symbolic boundary between “us”—the native members of the political community—and “them”—the immigrant outsiders. This boundary-making dimension is analytically significant for understanding how public support for unequal welfare distribution and labour market discrimination against immigrants is legitimised. Survey-based research has shown that subjective perceptions are more important than objective conditions in shaping chauvinist attitudes (Bell et al., 2023; Quandt and Schmidt, 2024). First, exclusionary preferences in welfare allocation are closely associated with perceptions that immigrants are undeserving of social support (Harell et al., 2021; Saar et al., 2022). Second, deservingness judgments are not solely based on instrumental considerations (e.g., perceived economic contribution), but also reflect cultural and identity-based criteria (van Oorschot et al., 2017; Harell et al., 2021). In particular, nationalism emerges as a highly relevant factor in explaining cultural as well as economic perceived threats (Callens and Meuleman, 2023). As Harell and colleagues pointed out: “What does matter – and matters a lot – is a sense of shared ‘we-ness’ which generates a commitment to belonging together, acting together, governing a territory, preserving a patrimony, sharing a public life and supporting each other in times of need” (Harell et al., 2021, p. 999).^1^ In other words, deservingness functions as a moral filter through which social solidarity is selectively applied, legitimising the exclusion of those perceived as external to the national “we.”

Within this framework, welfare and labour market chauvinism represent specific expressions of a broader *nativist* ingroup favouritism. Originally defined by Higham (1955, p. 4) as “an intense opposition to an internal minority on the ground of its foreign (that is, un-American) connections,” *nativism* has since been refined and distinguished from related notions such as racism, nationalism, and xenophobia (Betz, 2017; Guia, 2016). Despite its various forms—cultural, economic, racial, or secularist—most definitions share three elements. First, nativism draws a symbolic boundary between natives and foreigners, grounded in temporal distance (old vs. new inhabitants) and/or cultural distinctiveness (shared values and traditions). Second, foreignness is framed as a threat to the integrity of the nation. Third, this perceived threat legitimises preferential treatment for natives in the allocation of societal resources (Betz, 2022). The first two concern beliefs about the criteria for inclusion within the national ingroup. In contrast, the third reflects the motivation to safeguard the ingroup’s interests against perceived outgroups. At the individual level, this logic mirrors the mechanisms outlined by Social Identity Theory, including social categorisation, ingroup favouritism, and outgroup discrimination (Tajfel, 1981; Turner, 1987; Citrin and Sides, 2004). Referring to Social Identity Theory and in particular to the definition introduced by Marilynn Brewer, Citrin and colleagues (Citrin et al., 2001; Citrin and Sides, 2004) proposed a conceptualization of national identity based on three dimensions: (1) cognitive, that is, self-categorisation as a group member (who am I?); (2) affective, that is, the strength of emotional attachment; (3) normative, that is, beliefs about the criteria for inclusion in the group/the attributes of the prototypical member (who are we?).

The social categorisation based on cultural/temporal nativist differentiation may be better understood in terms of contents establishing the normative dimension of national identity, which refers to the norms, beliefs and values perceived as prototypical of group identity (Citrin et al., 2001; Citrin and Sides, 2004). This area of investigation is dominated by the widely criticised “ethnic/civic” dichotomy (Kohn, 1961; Smith, 1991). Despite the epistemological and methodological weaknesses (Brubaker, 1992; Shulman, 2002), the ethnic-civic dichotomy has been extensively utilised to investigate individual conceptions of nationhood/national symbolic boundaries in a comparative perspective. Large-scale surveys such as the ISSP National Identity modules (1995, 2003, 2013, 2023), the European Values Study (2017), and Pew’s Global Attitudes Survey (2016) typically ask respondents to evaluate the importance of attributes such as birthplace, ancestry, religion, language, citizenship, or respect for laws for being a “true” member of the nation (Mader and Mußotter, 2025). Empirical results rarely map neatly onto the rigid dichotomy derived from historical and legal traditions of nationalism (among many others, Jones and Smith, 2001; Reeskens and Hooghe, 2010; Guglielmi and Vezzoni, 2016; Ariely, 2020). In general, evidence from cross-national surveys indicates that civic and ethnic conceptions of national identity are neither mutually exclusive nor uniformly distributed, as many individuals simultaneously endorse elements of both (for reviews, see Larsen, 2017). Recently, four ideal-typical patterns of national boundary making across 42 countries were identified, addressing the multidimensionality of national identity (May, 2023). However, despite the theoretical, methodological, and empirical inconsistencies within the civic–ethnic dichotomy, the terminology retains heuristic value when applied analytically rather than prescriptively (Koning, 2011; Trittler, 2017; Ariely, 2020; Piwoni and Mußotter, 2023). In particular, using these instruments allows researchers to distinguish between elite conceptions of what the nation *is* or *ought to be* and citizens’ own understandings of national belonging (Helbling et al., 2016). Moreover, this approach makes it possible to identify recurrent attitudinal patterns within the population through the use of comparative statistical analyses.

Turning to the core focus of the article, an important issue in empirical research using this framework concerns the ambivalent role of civic conceptions of national identity on attitudes towards migration. While ethnic understandings of national boundaries seem to be consistently linked to exclusionary attitudes, civic ones display more context-dependent and variable effects (Tamir, 2019; Simonsen and Bonikowski, 2020; Lindstam et al., 2021; Indelicato and Martín, 2024). While survey research increasingly suggests that opposition to immigration is shaped more by cultural tensions based on national/ethnic identities, values and a lack of intergroup contact rather than by material concerns, this evidence remains theoretically and methodologically fragmented (Hainmueller and Hopkins, 2014; Schmidt and Quandt, 2018; Heath et al., 2020). The common denominator is the reference, in a more or less explicit way, to Social Identity Theory (SIT), but various psychological frameworks have been developed to explain attitudes toward immigrants. As elaborated in subsequent sections, this article specifically draws on Verkuyten’s (2009, 2018) Group Identity Lens model, which conceptualises national identity as a cognitive lens through which individuals interpret intergroup relations and evaluate social hierarchies. This approach is consistent with self-categorisation as a group member (“who am I?”) in line with the definition of national identity as social identity introduced by Citrin and colleagues (Citrin et al., 2001; Citrin and Sides, 2004).

Within this theoretical framework, the article seeks to advance existing research by analysing how civic and ethnic conceptions of national identity—together with feelings of national and supranational belonging—shape support for native employment priority, both directly and indirectly, through perceived threats and distrust of foreigners. The article is structured as follows. The next section outlines the theoretical framework and related hypotheses. The third section describes the data and methodological approach. The fourth presents empirical analysis, while the concluding section discusses the main findings, acknowledges limitations, and suggests avenues for future research.

## 2 Theoretical background

How do different conceptions of nationhood sustain or inhibit discriminatory attitudes, such as support for welfare or labour market chauvinism? A tentative answer can be outlined along three interrelated dimensions: conceptual, contextual, and psychological.

First, conceptually, the shared norms about the attributes of a “good” member of the nation, and its ethnic or civic definition, constitute what sociologists call *national symbolic boundaries*. Lamont and Molnár (2002: 168) define them as the “conceptual distinctions that separate people into groups and generate feelings of similarity and group membership.” Ethnic and national symbolic boundaries may be signalled through language, skin colour, clothing, lifestyles, or values, yet the salience of these markers is flexible: the same feature can be highlighted, downplayed, or ignored depending on the social interaction (Barth, 1969) and interpretative frames (Brubaker, 2004). In research on national identity, these *symbolic boundaries* resonate with broader theoretical debate on the origins of nations. Primordialist approaches regard nations as natural, enduring communities rooted in ancestry, religion, or territory (Kohn, 1944; Geertz, 1973). Constructivist perspectives, by contrast, emphasise the modern and socially constructed nature of nationhood, which is reproduced through institutions, political practices, and symbolic representations (Anderson, 1991; Gellner, 1983; Hobsbawm, 1990). Positioned between these poles, the ethno-symbolist perspective focuses on the lasting significance of myths, memories, and cultural traditions as symbolic resources for contemporary nation-building (Smith, 1986, 1991, 2000).

Situated against this backdrop, the ethnic–civic dichotomy (Kohn, 1961) has profoundly shaped research on nationalism and national identity. While civic nationalism is understood to accentuate voluntary political participation, institutional loyalty, and legal citizenship, ethnic nationalism foregrounds ascriptive characteristics such as ancestry, language, and cultural heritage. To move beyond the limits of the traditional ethnic-civic dichotomy, scholars have underscored the multidimensional character of nationhood. Eisenstadt and Giesen (1995) identified three symbolic codes of belonging: *primordial*, defining membership through ascribed traits such as lineage or ethnicity and allowing little inclusion; *cultural*, linking identity to sacred or transcendental orders—faith, reason, or progress—and permitting belonging through conversion; and *civil*, grounded in shared customs, institutions, and norms of conduct, often implicit, as in Billig’s (1995) “banal nationalism.” Kymlicka (2001) similarly distinguishes ethnic, cultural/traditional, and civic forms of belonging, arguing that modern nations combine inherited ties with participation in common institutions and civic values. Brubaker (1992, 2004) and Smith (2000) likewise reject rigid oppositions, emphasising the interplay of ethnic, cultural, and civic–political elements that take shape differently across historical and national contexts. More recently, Kaufmann (2017, 2019) introduced the notion of *ethnic majoritarianism* to describe how majority traditions endure within ostensibly civic narratives, shaping contemporary forms of exclusion. The term is used to denote the perceived cultural dominance of the national majority, which extends beyond descent-based criteria, while remaining closely connected to them.

Second, beyond this conceptual refinement highlighting the multidimensional character of nationhood, it is crucial to acknowledge that civic and ethnic elements frequently coexist within individuals and are shaped by contextual and situational factors. Ethnic components may remain latent and become salient under specific circumstances, whereas civic understandings, though formally inclusive, can function as subtle mechanisms of exclusion when immigrants are perceived as unwilling or unable to assimilate or internalise the host society’s “core values” (May, 2023; Lindstam et al., 2021; Ariely, 2020; Simonsen and Bonikowski, 2020; Tamir, 2019). The impact of these identity conceptions on exclusionary attitudes is highly context dependent. Their salience and direction are conditioned by institutional and political environments —such as migration regimes and elite discourses —that frame national belonging either inclusively, as “shared citizenship,” or defensively, as “defending our values” (van der Brug and Harteveld, 2021; Kaufmann, 2019). Empirical research across Europe consistently supports this contextual and dynamic interpretation. Comparative analyses of survey data show that *ethnic* conceptions of nationhood—grounded in ancestry, religion, or cultural homogeneity—are robustly associated with exclusionary attitudes toward immigrants, whereas *civic* understandings display far more heterogeneous effects (Larsen, 2017; May, 2023; Indelicato and Martín, 2024). Larsen’s (2017) work highlights a persistent East–West divide: in Central and Eastern Europe, ethnic nationalism is “mobilised” and strongly linked to anti-immigrant sentiment, while in Western Europe, civic frameworks prevail but remain vulnerable to ethnic reinterpretation. Similarly, May (2023) demonstrates that exclusionary orientations are not confined to ethnocultural forms of nationhood; in many Western contexts—such as France and Britain—*assimilationist* civic repertoires require adherence to dominant cultural norms, blurring the line between inclusion and exclusion. Indelicato and Martín (2024) identify a threefold structure—nationalism, cultural patriotism, and political patriotism—showing that nationalism and cultural pride consistently predict negative attitudes toward immigrants, while political (civic) patriotism fosters inclusion only in stable, high-trust environments.

Finally, the interaction between the symbolic content of national identity and the broader political context provides a framework for examining the psychological pathways through which national identity shapes attitudes towards immigrants. A common foundation lies in Social Identity Theory (Tajfel, 1981), which posits that individuals derive part of their self-concept from group memberships, fostering ingroup favouritism and outgroup derogation when social identity becomes salient. Building on this premise, Integrated Threat Theory (Stephan and Stephan, 2000, 2017) specifies that prejudice emerges from perceived realistic threats—such as competition for jobs or resources—and symbolic threats to cultural values and social cohesion, while Realistic Group Conflict Theory (Sherif, 1966) links hostility to actual or perceived intergroup competition. In contrast, Intergroup Contact Theory (Allport, 1954; Pettigrew and Tropp, 2006) offers a more optimistic perspective, suggesting that sustained, cooperative contact under conditions of equal status and institutional support can mitigate prejudice by fostering empathy and reducing perceived threat. Complementary perspectives within social psychology, notably Social Dominance Theory (Pratto et al., 2006) and System Justification Theory (Jost, 2020), extend the analysis to system-level motives, showing how preferences for hierarchy and stability reinforce exclusionary orientations. Together, these frameworks suggest that negative orientations toward immigrants arise from a dynamic interplay of identity processes, threat perceptions, hierarchy motives, and the wider intergroup social climate.

With respect to the present research question—how national identity influences support for labour market chauvinism—Verkuyten’s (2009, 2018) *Group Identity Lens* model offers a useful framework. Building on Social Identity Theory and Integrated Threat Theory, it argues that perceived threat mediates the relationship between national identity and ingroup favouritism, with group identity functioning as a “lens” through which individuals interpret intergroup relations and perceive outgroups as threatening. While much research has explored national identity and perceived threat separately, few studies have examined their joint effects, and most focus narrowly on the cognitive and affective dimensions of identity. By contrast, the normative dimension of national identity, referring to beliefs about what it means to be a “good” national member, has received far less attention (Huddy, 2001; Konings et al., 2023). This neglect may partly reflect measurement limitations, as few large-scale surveys capture both the strength of national attachment and the meanings ascribed to belonging and perceived threat (Ariely, 2020).

However, recent research has contributed to highlighting the role of mediating factors—such as perceived threat and empathy—in translating ethnic and civic conceptions of nationhood into intergroup attitudes. Across different contexts, these studies converge on the finding that the link between national identification and exclusionary orientations operates largely through threat-related processes. For instance, Brylka et al. (2015) showed that among majority-group members in Finland, stronger national identification increases perceptions of threat (and decreases perceived benefits) from immigration, which in turn predicts more negative attitudes towards immigrants. Using Canadian ISSP data, Sumino (2017) reported that ethnic identity heightens collective threat perceptions, whereas civic identity reduces them; these threat perceptions statistically mediate the effect of identity on support for multiculturalism. Employing both survey and experimental data from Hungary, Kende et al. (2018) demonstrated that endorsing an ethnic definition of citizenship heightens perceived threat and reduces empathy, thereby lowering support for pro-minority collective action, while civic conceptions reverse this pathway. Using ISSP data from 33 countries, Caricati (2018) provided cross-national evidence that perceived intergroup threat mediates the association between national identity and restrictive immigration attitudes.

Besides perceived threat, the mediating role of trust in linking national identity to attitudes toward immigrants has gained growing attention (Voci, 2006; Freitag and Kijewski, 2017). It is not surprising, given that trust and perceived threat form core components of the intergroup social climate (Dovidio et al., 2009; Schmid et al., 2014). Trust may be defined as a relational and normative expectation concerning others’ intentions and reliability (Newton and Zmerli, 2011), operating at both interpersonal and intergroup levels. Within the context of national identity, low outgroup trust often reflects perceptions that immigrants do not share the moral norms and civic values defining the national community, perceptions that reinforce symbolic threat and weaken social cohesion (Dinesen and Sønderskov, 2018). Conversely, higher levels of generalised and intergroup trust may foster openness to diversity by mitigating defensive reactions rooted in identity protection (Uslaner, 2012). Using ESS data from 23 European countries, Pellegrini et al. (2021) find that individuals who feel socially excluded exhibit lower generalised trust, which in turn predicts more negative attitudes toward immigrants.

Nevertheless, the relationship between national identity, trust, and attitudes towards immigrants is multifaceted. While certain studies indicate that increased ethnic diversity may undermine social trust due to exposure effects, there is compelling evidence that inclusive social norms and positive intergroup contact can attenuate or even counteract this dynamic (Schmid et al., 2014). Notably, empirical research seldom employs analytical frameworks in which both trust and perceived threat are modelled as simultaneous mediators linking national identity to anti-immigrant attitudes. However, the limited survey-based analyses that do investigate these dual mechanisms consistently support the conceptualisation of national identity as a distinctive interpretative “lens” shaping intergroup perceptions and responses. For example, individuals whose trust is predominantly limited to the ingroup tend to endorse more nativist immigration preferences, a tendency that is frequently intensified by heightened perceptions of collective threat (Crepaz et al., 2014). Guglielmi (2021), referring to the Italian case, found that ethnic national identity increases threat perceptions and diminishes outgroup trust, both of which jointly mediate support for anti-immigrant discrimination; interestingly, civic national identity influences such discrimination solely through threat perceptions, without involving the distrust pathway. Similarly, analysing data from ten Central and Eastern European countries, found that outgroup trust moderates the impact of ethnocultural national identity on immigration concerns, with low trust and exclusive identity definitions interacting to reinforce exclusionary attitudes.

### 2.1 The present study

To address these dynamics, this article examines how the ethnic and civic facets of national identity, together with the degree of national attachment, influence support for a specific exclusionary attitude—namely, the belief that, in circumstances of job scarcity, priority should be given to co-nationals—both directly and indirectly, through perceived threat and intergroup distrust.

Empirically, the analysis employs structural equation modelling (SEM) with survey data from the 2017 European Values Study, covering seven European countries. SEM is particularly suited to disentangling the direct and indirect pathways through which the symbolic boundaries of nationhood shape support for native employment priority, while testing the cross-national comparability of these constructs.

As Huddy (2001) observes, social identity processes rarely unfold in isolation from the institutional and political environments in which they are embedded, yet much psychological research tends to abstract them from these real-world contexts. It is acknowledged, however, that SEM primarily captures individual-level relationships and does not directly account for macro-contextual factors—such as migration regimes, party systems, or welfare institutions—that may condition how national identity impacts on attitudes. While it would be possible, in principle, to employ a multilevel SEM framework to incorporate such country-level effects, the present analysis deliberately focuses on individual-level variation to isolate the micro-mechanisms linking identity-based perceptions to exclusionary preferences. Accordingly, the study offers a baseline model of these processes while acknowledging that their strength and configuration may differ across national settings.

Two main hypotheses are tested, consistently with the “Group Identity Lens” approach presented before:

*H1*. Nativist Spiral Hypothesis: Ethnic-majoritarian conceptions of national identity are expected to influence support for native employment priority both directly and indirectly, by reinforcing perceptions of threat and intergroup distrust.

*H2*. Two-Faced Civility Hypothesis: While civic conceptions of national identity are anticipated to reduce distrust and directly decrease support for native priority, they may simultaneously exert an indirect positive effect on exclusionary preferences by amplifying perceived cultural and economic threats.

## 3 Method

**Figure 1:**
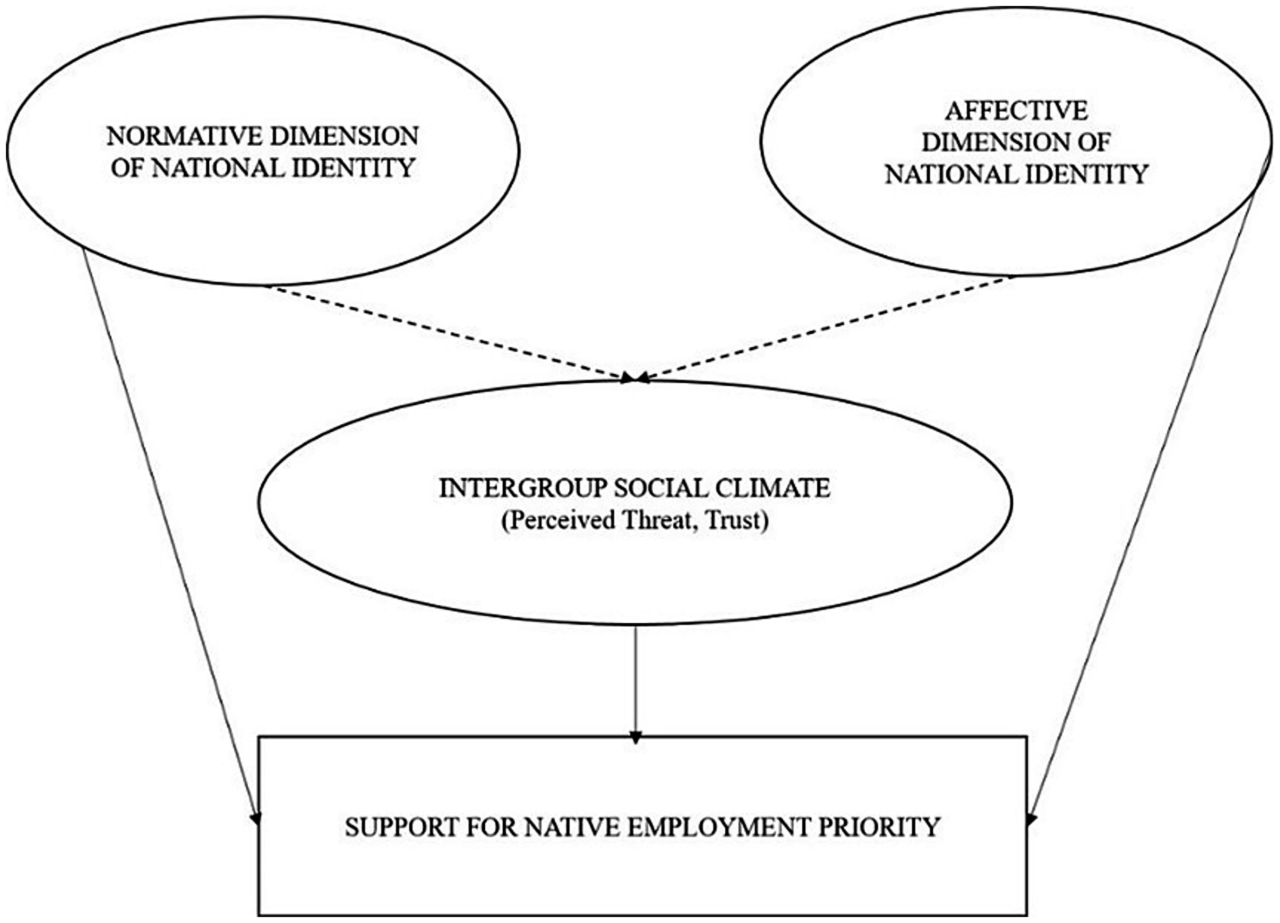
The NITT model (National Identity – Threat – Trust) (Guglielmi, 2021).

Drawing on the theoretical considerations outlined above, this study proposes the National Identity Threat Trust (NITT) model. The simplified version of the model is in Figure 1. The NITT model should not be regarded as an entirely new theoretical construct but rather as an empirical framework designed to integrate and extend established approaches. Its main contribution lies in the systematic integration of multiple dimensions of national identity—both normative and affective—with perceived threat and intergroup trust within a single, empirically testable structure. This design enables comparative analysis and facilitates the assessment of measurement invariance across the seven European countries included in the study.

The empirical analysis draws on data from the last wave of the European Values Study, a large-scale, cross-national research programme that explores citizens’ ideas, beliefs, preferences, attitudes, and values across Europe. Conducted approximately every 10 years since 1981, it provides repeated cross-sectional data that enable both longitudinal and comparative analyses of social change. While the early waves included a limited number of countries, the project has progressively expanded its coverage. The fifth wave (2017–2020), which provides the empirical basis for this study, includes 37 participating countries. The questionnaire for this wave contains detailed items on national identity—including both the “true co-national” battery and measures of attachment—as well as questions on perceived threat and trust. These harmonised, high-quality data make the EVS a unique resource for examining how values and attitudes—particularly those concerning national identity and perceptions of immigrants—vary across diverse European contexts.

Analysis in this article focuses on seven EU countries in different regional areas: Central Europe (France, Germany), the Anglo-Saxon world (Great Britain), Central-Eastern Europe (Hungary, Poland), and Southern Europe (Portugal, Italy).^2^ The seven European countries present varying welfare regimes and migration contexts, enabling a broader comparative assessment. From the perspective of migration regimes, France, Germany, and Great Britain represent long-established immigration countries with developed integration policies, whereas Italy and Portugal are more recent destinations, and Hungary and Poland remain predominantly emigration contexts with limited immigration until the mid-2000s. In terms of citizenship models, France exemplifies a republican tradition grounded in universalist ideals and ius soli elements, while Germany—though historically ius sanguinis—has reformed towards a more civic-integrative model. The UK maintains a liberal-pluralist approach with strong multicultural precedents, though more recently challenged. Conversely, Italy, Poland, and Hungary adhere more closely to descent-based, ethnic understandings of citizenship, with limited civic integration channels. Portugal stands out for its colonial legacy and relatively liberal naturalisation policies, despite a weak institutional emphasis on civic integration. These contrasts provide a valuable basis to examine how national identity configurations influence public attitudes toward labour market inequalities and nativistic discrimination.

Furthermore, the countries show differing levels of public support for native employment priority. Germany reports the lowest level of agreement (30%) with the statement “When jobs are scarce, employers should give priority to [NATIONALITY] people over immigrants,” while Hungary shows the highest agreement (84%), followed by Italy (67%) and Poland (62%). Great Britain (39%) and France (43%) fall in between (EVS, 2022).

**Figure 2:**
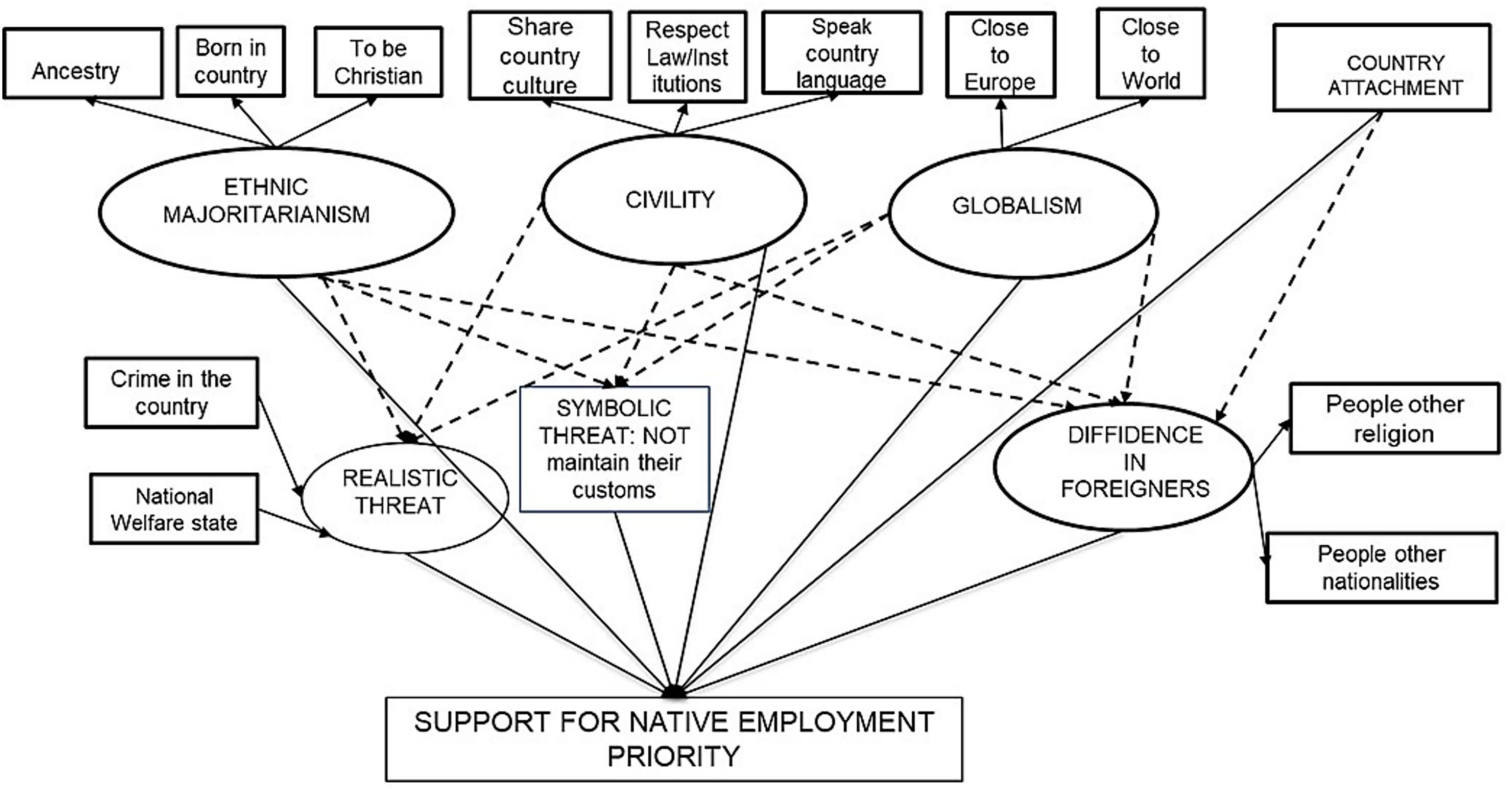
Full NITT SEM model (National Identity – Threat – Trust). Circles represent latent variables and squares represent observed variables. Single-headed arrows represent “causal” effects. Solid lines represent direct effects. Dotted lines represent paths from identity components to natives favouritism mediated by intergroup social climate (threats and outgroup trust). Covariances between independent variables and between mediators are estimated, but not represented for space reasons.

The full model combines a measurement model with five latent constructs—ethnic majoritarianism, civility, globalism, realistic threat, symbolic threat and distrust—and a structural model capturing direct and indirect effects on support for native employment priority. Affective and normative dimensions of national identity are hypothesised to shape Support for native employment priority via perceived threat and distrust. Covariances are estimated among identity and attitudinal constructs where directionality is not assumed (see Figure 2).

Measures included in the model are as follows.

### 3.1 Support for native employment priority

Support for the idea that natives should have priority in the event of competition for a job is measured by an observed variable, the level of agreement with the following statement: “When jobs are scarce, employers should give priority to [NATIONALITY] people over immigrants.” (5-point scale: “1 = strongly agree to 5 = strongly disagree”).

### 3.2 National identity

a) Normative dimension of national identity: As regards the normative dimension of national identity, the model distinguishes between the ethnic majoritarian code of national belonging and civility. Both latent variables are measured by assessing the extent to which a respondent considers a list of attributes important to be a “true” co-national. The ethnic majoritarian code is measured by a list of symbolic boundaries defining a temporal and values-based differentiation: Being born in [respondent’s country], Having [respondent’s country] ancestry, and Being Christian. The civility code implies more inclusive and achievable attributes: Being able to speak the national language, Sharing [respondent’s country] culture, and Respecting [respondent’s country] political institutions and laws (4-point scale 4 = very important to 1 = not at all; reverse score).
b) Affective dimension of national identity: To take into account the distinction between exclusive identification vs. multiple territorial identities, the model includes a latent variable (globalism) measuring the level of attachment to Europe and the world and an observed variable concerning the level of closeness to country (4-point scale 4 = very close to 1 = not at all; reverse score).

### 3.3 Intergroup social climate (threats and trust)

a) Perceived collective threat

*Realistic threat* is measured by a latent variable based on two item, respectively measuring the level of agreement with the idea that migration is a burden for the national welfare state and is responsible for the increase in the rate of crime in Italy (self-rating on a scale from 1 to 10, where Immigrants do not make crime problems worse (10)/Immigrants make crime problems worse (1); reverse score; Immigrants are a strain on a country’s welfare system (10)/Immigrants are not a strain on a country’s welfare system (1), reverse score).^3^

As regards *symbolic threat,* the model includes a proxy variable, self-rating on a scale from 1 to 10, where 1 is “It is better if immigrants maintain their distinct customs and traditions” and 10 is “It is better if immigrants do not maintain their distinct customs and traditions.” Even if it is not a proper measure of symbolic threat, it is reasonable to suppose that people not comfortable with the idea that migrants maintain their customs (and then opt for assimilation) are more sensitive to symbolic threat. Scholars habitually use this item to measure the adoption of a multicultural/assimilationist view in intergroup relations.

b) Distrust of foreigners

To measure trust in foreigners, the NITT model includes a latent variable based on the level of trust in people of another religion and people of another nationality (both scaled 1–4).

## 4 Results

### 4.1 The full NITT SEM model

Figure 2 shows the full structural equation model tested.^4^

The model demonstrates strong empirical validity across the pooled dataset, with excellent fit indices (RMSEA = 0.046, CFI = 0.970, TLI = 0.951, SRMR = 0.030).^5^

Ethnic majoritarianism and civility are positively correlated (*β* = 0.614), indicating that—consistent with previous research—the two conceptions of nationhood are not mutually exclusive but partly overlapping. Ethnic conception of national identity thus appears to introduce an exclusionary boundary into an otherwise broadly shared civic–cultural understanding of belonging. The mediating variables—symbolic threat, realistic threat, and intergroup distrust—are moderately interrelated, confirming that perceived threats and distrust represent distinct yet mutually reinforcing mechanisms. The strongest correlation is observed between realistic threat and distrust (*β* = 0.244, *p* < 0.001), followed by realistic and symbolic threat (*β* = 0.176, *p* < 0.001), while the association between symbolic threat and distrust is comparatively weak (*β* = 0.082, *p* < 0.001). Both ethnic-majoritarian and civic conceptions of national identity significantly increase perceived realistic threat (*β* = 0.288, *p* < 0.001; *β* = 0.178, *p* < 0.001, respectively), whereas cosmopolitan orientation exerts the opposite influence, reducing such perceptions (*β* = −0.170, *p* < 0.001). Civic identity also heightens symbolic or cultural threat (*β* = 0.238, *p* < 0.001)—that is, stronger agreement with the view that immigrants should not maintain their own customs—which in turn predicts greater support for native employment priority (*β* = 0.067, *p* < 0.001). Ethnic-majoritarian national identity conception, by contrast, slightly reduces this form of cultural threat when other paths are controlled (*β* = −0.035, *p* = 0.022). A similar pattern emerges for intergroup distrust: ethnic-majoritarian identity markedly increases distrust (*β* = 0.285, *p* < 0.001), whereas both civility (*β* = −0.053, *p* = 0.002) and globalism (*β* = −0.180, *p* < 0.001) significantly reduce it. Attachment to one’s country shows no meaningful effect on either perceived realistic threat or intergroup distrust. Overall, these results indicate that exclusionary understandings of national identity activate both threat and distrust mechanisms, whereas more inclusive, cosmopolitan orientations are associated with lower perceived threat and greater intergroup trust. Overall, the pattern of relationships reveals that exclusionary understandings of nationhood consistently amplify perceptions of competition and social distance, whereas civic and cosmopolitan orientations partly mitigate distrust but not perceived threat.

### 4.2 The nativist spiral and the two-faced civility hypotheses

The results provide strong support for the *Nativist Spiral* hypothesis, anticipating both direct and indirect effects of ethnic-majoritarian conception of national identity on native employment priority.^6^ Ethnic-majoritarian identity exerts a substantial total effect on support for native employment priority (*β* = 0.438, *p* < 0.001), composed of both a robust direct component (*β* = 0.326, *p* < 0.001) and significant indirect pathways. The total indirect effect (*β* = 0.111, *p* < 0.001) confirms that perceived threats and distrust jointly reinforce the association between national identity and exclusionary attitudes. Among the mediators, realistic threat emerges as the most influential pathway (*β* = 0.084, *p* < 0.001), followed by symbolic threat (*β* = 0.030, *p* < 0.001). Intergroup distrust has a small negative indirect effect (*β* = −0.002, *p* = 0.030), suggesting that, once threat perceptions are considered, distrust does not independently amplify nativist orientations. Overall, these findings indicate that ethnic conceptions of nationhood foster exclusionary labour market preferences through a dual mechanism: a direct normative endorsement of ingroup favouritism and, to a lesser extent, an indirect reinforcement via perceived economic and cultural threats.

The *Two-Faced Civility* hypothesis is partially supported, as the civic conception of national identity shows mixed effects. Its direct effect on support for native employment priority is weak but positive (*β* = 0.053, *p* < 0.001), implying a slight increase in exclusionary support, net of mediators. At the same time, civility reduces intergroup distrust (*β* = −0.053, *p* = 0.002) but increases perceived realistic threats (*β* = 0.178, *p* < 0.001) and symbolic/cultural (*β* = 0.238, *p* < 0.001), and both threats are positively associated with native priority. As a result, the total effect of civility on native priority is positive (*β* = 0.116, *p* < 0.001), with a total indirect effect of *β* = 0.062 (*p* < 0.001) driven mainly by realistic threat (*β* = 0.052, *p* < 0.001) and, to a lesser extent, symbolic/cultural threat (*β* = 0.016, *p* < 0.001). Intergroup distrust contributes a small negative mediation (*β* = −0.005, *p* = 0.003), consistent with civility lowering distrust. Taken together, these patterns underline the ambivalence of civic national symbolic boundaries: while it modestly dampens distrust, it heightens sensitivity to economic and cultural threat and even directly carries a faint exclusionary imprint.

### 4.3 Cross-country comparison

Building on the strong fit of the NITT model in the pooled dataset, the analysis proceeds to a country-specific comparison to assess how national identity influences support for native employment priority across different national contexts. To preliminarily ensure the comparability of latent constructs across countries, a Multi-Group Confirmatory Factor Analysis (MGCFA) was conducted to test for measurement invariance at the configural, metric, and scalar levels. Both configural and metric invariance were supported. The configural model showed strong fit indices (CFI = 0.982; RMSEA = 0.041; SRMR = 0.030), and the metric model remained within acceptable thresholds (ΔRMSEA = 0.009; ΔSRMR = 0.020), despite a slightly elevated ΔCFI (0.014 vs. the 0.010 cut-off). Scalar invariance, however, was not supported (CFI = 0.867; RMSEA = 0.097; SRMR = 0.084). These findings confirm that the structural paths can be meaningfully compared across countries, though comparisons of latent means should be avoided. In sociological terms, metric invariance indicates that the structure and relative salience of the boundary criteria are shared across contexts, whereas the lack of scalar invariance reveals that their reference points remain country-specific. Built on this findings, the following analysis employed a multi-group (cross-national) SEM, factor loadings for all latent constructs were constrained to equality across countries, while all structural paths were freely estimated.^7^ Both the measurement and structural components were estimated simultaneously.

First, multi-group analysis revealed that the correlation between ethnic-majoritarian and civil conceptions of national identity is comparatively weak in France (*β* = 0.474, *p* < 0.001), Italy (*β* = 0.465, *p* < 0.001), suggesting that civic and ethnic symbolic boundaries are somewhat distinct or even in tension. By contrast, in Poland (*β* = 0.802, *p* < 0.001) and Portugal (*β* = 0.799, *p* < 0.001), the link is powerful, implying that civic national boundaries are more tightly intertwined with ascribed elements. Hungary (*β* = 0.570, *p* < 0.001), Great Britain (*β* = 0.601, *p* < 0.001) and Germany (*β* = 0.691, *p* < 0.001) occupy an intermediate position.

Second, the country-specific configuration of relationships between national identity, perceived threats, intergroup trust and support for native employment priority emerges (Table 1).

**Table 1:** Cross-national patterns of ethnic and civic identity effects on native employment priority.

Country	Correlation between ethnic-majoritarianism and civility (*β*)	H1 Nativist spiral	H2 Two-faced civility	Ethnic-majoritarianism effect type	Civility effect type	Key mediation mechanisms
France	Low <=0.49	Yes	Yes	Direct and indirect	Direct and indirect	Realistic threat (H1/H2), Intergroup distrust (H1)
Germany	Moderate 0.50–0.70	Partially (direct only)	Partially (indirect only)	Direct only	Indirect only	Realistic threat (H2), Cultural threat (H2)
Great Britain	Moderate 0.50–0.70	Yes	Yes	Direct and indirect	Direct and indirect	Realistic threat (H1/H2), Cultural threat (H2)
Hungary	Moderate 0.50–0.70	Partially (indirect only)	Yes	Indirect only	Direct and indirect	Realistic threat (H1/H2)
Italy	Low <=0.49	Yes	Partially (indirect only)	Direct and indirect	Indirect only	Realistic threat (H1-H2), Intergroup distrust (H1), Cultural threat (H2)
Poland	High >0.70	Yes	No	Direct and indirect	None	Realistic threat (H1), Intergroup distrust (H1)
Portugal	High >0.70	Yes	No	Direct and indirect	None	Realistic threat (H1)

The *Nativist Spiral* hypothesis (H1) displays strong cross-national consistency, being supported in all countries examined. In line with expectations, in all cases except Germany and Hungary the ethnic-majoritarian conception of national identity exerts both direct and indirect effects on support for native employment priority, simultaneously heightening perceptions of threat and eroding intergroup trust. In Germany, the relationship between ethnic-majoritarian identity and support for native employment priority is predominantly direct, whereas in Hungary it operates mainly through indirect channels. Realistic (economic) threat emerges as the most consistent mediator, present in every country except Germany. Intergroup distrust acts as an additional mediator in France, Italy, and Poland.

By contrast, the *Two-Faced Civility* pattern (H2) is not supported in Portugal and Poland, where civic understandings of nationhood show no association—direct or indirect—with exclusionary attitudes. In the remaining countries, however, the pattern is context-dependent. It combines both direct and indirect effects in France, Great Britain, and Hungary, and appears only indirectly—via heightened perceptions of threat—in Germany and Italy. Indirect effects through realistic (economic) threat are consistently found in all countries where H2 holds, while cultural threat acts as an additional mediator in Germany, Great Britain, and Italy (Table 1).

While Table 1 offers a comparative overview, the following sections provide a more detailed interpretation of the empirical patterns in each national context.^8^

### 4.4 France

The French data strongly support the “Nativist Spiral” hypothesis (H1). The total effect of the ethnic-majoritarian conception on native employment priority is robust and significant (*β* = 0.417, *p* < 0.001). The impact is to a large extent direct (*β* = 0.271, *p* < 0.001), but also indirect via realistic threat (*β* = 0.114, *p* < 0.001) and to a lesser extent via intergroup distrust (*β* = 0.034, *p* < 0.001). The cultural threat path is modest and non-significant, indicating that economic and trust-based concerns dominate.

The Two-Faced Civility hypothesis (H2) is also supported, though it reveals the ambivalence of the civic conception of national identity. Civility has a positive total effect on exclusionary preferences (*β* = 0.210, *p* < 0.001), due to a sizeable direct effect (*β* = 0.114, *p* < 0.001) and a smaller indirect effect is (*β* = 0.096, *p* < 0.001). It is primarily via realistic threat (*β* = 0.084, *p* < 0.001), with very little contribution from cultural threat (*β* = 0.012, *p* = 0.048) and none from distrust.

To sum up, while ethnic symbolic boundaries foster exclusionary attitudes, directly and indirectly, *Civility* does not operate as a straightforwardly inclusive frame. Its net association with native-priority is positive, driven above all by a sizeable direct component and by a smaller pathway through realistic (economic) threat. This pattern suggests that civic criteria are being mobilised less as an antidote to exclusion than as a norm-conformist, boundary-defending logic linked to perceptions of competition and scarcity, rather than to cultural distance or social mistrust.

### 4.5 Germany

In Germany, the “Nativist Spiral” hypothesis (H1) receives partial support. The ethnic-majoritarian conception of national identity has a significant direct effect on support for native employment priority (*β* = 0.224, *p* < 0.001), but indirect effects are minimal and not significant (*β* = 0.016, *p* = 0.674). Only intergroup distrust mediates weakly (*β* = 0.021; *p* < 0.05), while realistic and cultural threat paths are non-significant. This suggests that an ethnic-majoritarian conception of national identity shapes exclusionary preferences primarily through direct associations, with little reinforcement via perceived threat or distrust.

Similarly, the “Two-Faced Civility” hypothesis (H2) is partially supported. Civility has a positive total effect (*β* = 0.228, *p* = 0.003), driven almost entirely by indirect pathways (*β* = 0.218, *p* < 0.001), especially via realistic threat (*β* = 0.170, *p* < 0.001) and to a lesser extent cultural threat (*β* = 0.037). The direct effect is negligible and not significant (*β* = 0.009, *p* = 0.913). These findings reveal the ambivalent nature of civic national identity in Germany: while nominally inclusive, it indirectly fuels exclusionary attitudes by heightening threat perceptions. Thus, civic identity in Germany behaves in a “two-faced” manner, symbolically open but practically capable of reinforcing defensive preferences toward immigration.

### 4.6 Great Britain

British data support the “Nativist Spiral” hypothesis (H1). Ethnic-majoritarian conception of national identity significantly predicts support for native employment priority (total *β* = 0.280, *p* < 0.001), combining a direct effect (*β* = 0.171, *p* < 0.001) with a substantial indirect effect (*β* = 0.109, *p* < 0.001). The key mediator is realistic threat (*β* = 0.100, *p* < 0.001), pointing to economic competition as the main channel linking ethnic national symbolic boundaries to exclusionary attitudes. Indirect effects via distrust and cultural threat are minimal or non-significant.

The “Two-Faced Civility” hypothesis (H2) is also supported. Civility exerts a positive total effect (*β* = 0.239, *p* < 0.001), with both direct (*β* = 0.170, *p* < 0.001) and indirect (*β* = 0.069, *p* < 0.001) components. The main indirect route is again realistic threat (*β* = 0.059, *p* < 0.001), with a smaller contribution from cultural threat (*β* = 0.015, *p* < 0.05). Distrust plays no meaningful role. These findings show that, in the UK, civic identity may simultaneously express openness while indirectly sustaining nativist attitudes through concerns about national cohesion and economic security.

### 4.7 Hungary

In Hungary, the “Nativist Spiral” hypothesis (H1) is partially confirmed, supported primarily through indirect effects. While the total effect of ethnic-majoritarianism on support for native employment priority is significant (*β* = 0.142, *p* = 0.001), the direct effect is not (*β* = 0.030, *p* = 0.491). The relationship is driven by indirect mechanisms (*β* = 0.112, *p* = 0.001), with realistic threat as the dominant mediator (*β* = 0.103, *p* < 0.001). Cultural threat and intergroup distrust are not significant. These findings suggest that economic concerns, rather than symbolic or trust-based fears, are the key drivers linking ethnic conception of national identity to exclusionary preferences in Hungary.

Support for the “Two-Faced Civility” hypothesis (H2) is clearly evident. Civility shows a significant total positive effect on support for native job priority (*β* = 0.169, *p* < 0.001), driven by both direct (*β* = 0.130, *p* = 0.002) and, to a lesser extent, indirect (*β* = 0.039, *p* < 0.05) components. The primary indirect path again runs through realistic threat (*β* = 0.033, *p* < 0.05), while cultural threat plays a minor, non-significant role, and distrust is negligible.

Substantively, these results suggest that, in the Hungarian context, both ethnic and civic frames become boundary-defending when linked to perceptions of economic competition and scarcity, rather than to cultural distance or social mistrust.

### 4.8 Italy

The Italian case strongly supports the “Nativist-Spiral” hypothesis (H1). Ethnic-majoritarian conception of national identity significantly predicts support for native employment priority (*β* = 0.375, *p* < 0.001), through both a direct (*β* = 0.237, *p* < 0.001) and an indirect effect (*β* = 0.138, *p* < 0.001). The main mediating factor is realistic threat (*β* = 0.104, *p* < 0.001), followed by intergroup distrust (*β* = 0.027, *p* < 0.001) and cultural threat (*β* = 0.007, *p* < 0.05).

For the “Two-Faced Civility” hypothesis (H2), the evidence is more mixed. Civility shows no significant total (*β* = 0.019, *p* = 0.512) or direct effect (*β* = −0.026, *p* = 0.321), suggesting no clear link with exclusionary attitudes. However, the indirect effect is small but significant (*β* = 0.044, *p* = 0.003), largely via realistic (*β* = 0.040, *p* = 0.001) and less via cultural threat (*β* = 0.008, *p* = 0.009), while the distrust path is not significant. This supports the “two-faced civility” hypothesis: although civic identity appears inclusive on the surface and fosters inclusive labour market relations, it may contribute to reinforcing the feeling that the nation is threatened by immigrants, a perception that largely fuels support for using nativist criteria if jobs are scarce.

### 4.9 Poland

Polish data offer strong support the “Nativist-Spiral” hypothesis (H1). Ethnic-majoritarian conception of national identity significantly predicts support for native employment priority (*β* = 0.447, *p* < 0.001), with both a substantial direct effect (*β* = 0.280, *p* < 0.001) and a notable indirect effect (*β* = 0.167, *p* < 0.001). The main mediating channel is realistic threat (*β* = 0.113, *p* < 0.001), followed by intergroup distrust (*β* = 0.057, *p* < 0.001), while cultural threat is non-significant. These findings suggest that economic and security-related concerns, rather than symbolic threats, primarily link ethnic conceptions of national symbolic boundaries to exclusionary job preferences.

Conversely, the “Two-Faced Civility” hypothesis (H2) receives no support. Civic conception of national identity has no significant effect on support for native employment priority, either directly (*β* = −0.002, *p* = 0.973) or indirectly (*β* = −0.041, *p* = 0.201). None of the mediating pathways—realistic threat, cultural threat, or distrust—reach statistical significance. These results indicate that in Poland, civic identity is largely neutral: it neither directly fosters inclusive attitudes nor contributes to reinforcing the perception of threat or diffidence. Furthermore, the very high correlation between civic and ethnic identity (*β* = 0.80) suggests that, although modelled as distinct latent constructs, they reflect a largely unified national schema. In this sense, civic identity represents less an independent source of attitudes than a symbolic extension of the ethnic boundary logic that dominates the national field.

### 4.10 Portugal

Portuguese data support the “Nativist Spiral” Hypothesis (H1). Ethnic-majoritarian conception of national identity significantly predicts support for native employment priority (*β* = 0.252, *p* < 0.001), with both a direct effect (*β* = 0.143; *p* < 0.05) and a notable indirect effect (*β* = 0.109, *p* < 0.001). The dominant mediating pathway is realistic threat (*β* = 0.117, *p* < 0.001), while cultural threat and intergroup distrust have no significant impact. This suggests that exclusionary job preferences in Portugal are primarily driven by economic threat perceptions linked to an ethnic conception of national identity, rather than symbolic or trust-based concerns.

In contrast, the “Two-Faced civility” hypothesis does not find support in this case. H2 is not supported. Civic national identity shows no significant total (*β* = 0.067, *p* = 0.304), direct (*β* = 0.059, *p* = 0.342), or indirect (*β* = 0.008, *p* = 0.764) effects on support for native employment priority. Among the mediators, only distrust shows a marginally significant effect (*β* = 0.026, *p* = 0.038), though this is offset by a non-significant negative path through realistic threat. Cultural threat remains negligible. Overall, civic identity in Portugal appears weak and inconsistent in its influence, lacking clear evidence of either inclusive or exclusionary tendencies. It is worth noting that, as in Poland, the correlation between civic and ethnic identity, despite distinct latent variables, is very high (*β* = 0.79).

## 5 Discussion

Public support for restricting immigrants’ access to welfare and employment—often described as welfare or labour market chauvinism—has become a salient political issue across Europe (Bell et al., 2023; Betz, 2022). This article investigated how national symbolic boundaries shape these exclusionary preferences, focusing on the belief that native citizens should be prioritised over immigrants when jobs are scarce. The core premise, drawing on the Group Identity Lens model (Verkuyten, 2009, 2018), is that national identity functions as a perceptual lens through which social inequalities between natives and immigrants are both interpreted and legitimised. Analysis employing structural equation modelling on the EVS 2017 dataset across seven European countries substantiated that threat perceptions and trust serve as critical mediating mechanisms, with the capacity to transform seemingly inclusive civic norms into exclusionary attitudes. This interpretation aligns with prior research demonstrating that perception of threat may operate as a legitimising instrument for social stratification, yet its influence is neither uniform across European contexts nor detached from the specific cultural environments and narratives that shape individuals’ worldviews (Davidov et al., 2020; Ariely, 2024). Even ostensibly civic repertoires can embed implicit norms of cultural conformity, while ethnic boundaries may vary in their permeability depending on national narratives and integration regimes (Ariely, 2024).

Findings revealed a pronounced Nativist Spiral mechanism across all seven countries investigated, France, Germany, Great Britain, Hungary, Italy, Poland, Portugal.

Ethnic-majoritarian conceptions of nationhood consistently predict endorsement of native job preference, operating both directly and indirectly through elevated realistic, and to a lesser extent, symbolic threat perceptions alongside diminished outgroup trust. This suggests that when national belonging is normatively framed around majority traditions and ascribed boundaries, the identity lens interprets intergroup relations as inherently competitive and insecure, thereby rendering labour-market chauvinism a seemingly rational response. Second, findings confirm the ambivalent nature of civic national identity (Kaufmann, 2019; Simonsen and Bonikowski, 2020; Lindstam et al., 2021; May, 2023), highlighting one of the mechanisms underlying this apparent paradox: civic conception of national identity can dampen distrust yet simultaneously intensify perceptions of threat, thereby sustaining exclusionary preferences through direct and/or indirect pathways. Overall, its net impact on support for native employment priority is positive, suggesting that even ostensibly civic conceptions of belonging can carry a subtle exclusionary dimension when intertwined with perceptions of threat and competition. In particular, civic conceptions of national identity are positively associated with the idea that it is better that migrants do not maintain their specific customs. The findings reflect the ongoing scholarly debate on multiculturalism in liberal democracies, its alleged decline, and the rise of civic integration models grounded in post-national and universal liberal ideals (Joppke, 2017). Yet, empirical implementation often undermines these liberal principles, reinstating the native majority’s authority to define national culture (Larin, 2020; Mouritsen et al., 2019). This is particularly evident in European integration tests, which condition the acquisition of rights on unilateral evaluations of language, cultural, and historical knowledge (Kostakopoulou, 2010; Hackl, 2022).

Furthermore, the cross-national patterns observed for the *Two-Faced Civility* hypothesis underscore that the ambivalence of civic nationalism is contextually refracted through distinct historical and institutional lenses. This conditional inclusiveness is especially visible in France, Great Britain, Hungary and Germany (and to a lesser extent in Italy), while it is negligible in Portugal and Poland. Despite France and Britain are considered common examples of the “civic” model, several scholars stressed how French republican tradition emphasises secular and linguistic unity as *preconditions* for belonging and integration (Bugge, 2022; Fargues et al., 2024), while Britain’s multicultural legacy has been increasingly reframed around integration “duties” (Kaufmann, 2019). At the individual level, Simonsen and Bonikowski (2020), for example, observed that British civic ideals, though formally inclusive, often function as *conditional* markers of membership, particularly in discourses surrounding Muslim minorities. In Hungary, too, analysis revealed that civic identity plays a key role in legitimating inequalities against migrants in the labour market. However, the context here differs from that in France or Great Britain (Barna and Koltai, 2019; Kende et al., 2018), as it is a typical case of mobilised ethnic nationalism (Larsen, 2017).

In Germany and Italy, the civic conception of national identity affects support for native employment priority only indirectly through perceived threat, stronger in Germany, weaker in Italy. Once an ethno-cultural nation with exclusive membership (Brubaker, 1992), Germany’s post-war discourse increasingly promoted civic identity alongside liberalised citizenship and immigration policies (Mader et al., 2021). The indirect effect observed here aligns with survey experiments indicating that many Germans endorse both civic and ethno-cultural conceptions of nationhood, resulting in attitudinal ambivalence and volatility toward immigration (Lindstam et al., 2021). In contrast to Germany, Italy has only recently experienced significant immigration flows and maintains a highly restrictive citizenship law. The country is marked by a weak civic political culture and a fragmented ethno-cultural sense of national belonging. Italy represents a hybrid context in which civic and ethnic repertoires of national identity coexist and can be selectively reactivated as cultural defence mechanisms during periods of perceived insecurity (Antonsich, 2016; Guglielmi, 2021). By contrast, in Poland and Portugal any effect of civic conception of national identity on support for native priority employment was found, either direct or indirect. These findings resonate with the description of particular national narratives in these countries. In Poland, civic identity is largely inert amid a dominant ethnocultural narrative, or as Larsen called “stable mobilized ethnic nationalism” (Larsen, 2017:991). In Portugal, civic identity remains genuinely inclusive yet politically inert, as the lusotropicalist national narrative and the depoliticisation of immigration (Almeida and Corkill, 2015). make civic conceptions ineffective in shaping electoral preferences on migrant integration.

This contextual variability encapsulates the essence of *Two-Faced Civility*—a civic nationalism that can both extend and restrict the moral boundaries of solidarity, depending on the historical and political lens through which it is refracted. The implications of these results are twofold. Analytically, this research advances understanding beyond the traditional civic-ethnic binary by pinpointing threat and trust as functional pathways through which national identity moulds exclusionary preferences, while also emphasising how these mechanisms may be conditioned by national narratives and institutional context. Substantively, the findings reveal the limitations of policy approaches that rely predominantly on civic values, which may inadvertently reinforce symbolic boundaries and exclusion when such appeals are culturally constrained or securitised, thereby reproducing symbolic closure under the guise of inclusion (Mouritsen et al., 2019; Larin, 2020; Hackl, 2022).

The comparative design has made it possible to identify recurrent mechanisms linking national identity, perceived threat, and support for native employment priority, thereby providing robust evidence for the micro-level dynamics discussed in the introduction. Nevertheless, the analysis remains confined to individual-level relationships, relying primarily on descriptive contrasts between national cases. This approach does not fully capture how broader structural factors—such as migration and citizenship regimes, welfare state models, party systems, or the strength of populist radical right actors—may shape or moderate these relationships. Similarly, the findings only hint at the crucial role of discursive and institutional conditions under which identity frames are politicised and strategically mobilised by elites and media actors (Eberl et al., 2018; May and Czymara, 2024), without directly examining them. This represents an important limitation that future research should address by moving beyond the enduring dichotomy between civic and ethnic nationalism (Piwoni and Mußotter, 2023). From a methodological standpoint, this research agenda presents considerable challenges. It calls for the integration of survey-based attitudinal data with analyses of discursive repertoires, media agendas, and political communication processes operating across different analytical levels and temporal scales.

Ultimately, understanding these interpretive dynamics is crucial not only for explaining public support for exclusionary policies but also for unpacking the symbolic mechanisms that sustain broader patterns of legitimisation of social inequality, by obscuring systemic discrimination.

## Data Availability

Publicly available datasets were analyzed in this study. This data can be found at: EVS (2022).

## Footnotes

1 However, the same sense of collective “we-ness” can also acquire a narcissistic dimension, fostering defensive and self-congratulatory forms of nationalism (Marchlewska et al., 2020). This dynamic may further promote practices such as whataboutism or the diffusion of responsibility, providing moral justifications for collective inaction—for instance, in response to climate change (Kulin, 2025).

2 Previous application of the NITT model on the Italian case is in Guglielmi (2021).

3 The European Values Study (EVS) includes an additional item measuring economic threat—“Immigrants take jobs away from [NATIONALITY]/do not take away”—which was not included in the analysis in order to avoid conceptual and statistical overlap with the dependent variable, which concerns the prioritisation of jobs for co-nationals in times of economic scarcity.

4 In this article, SEM is performed using Mplus 8.1 software. To deal with missing data, Full Information Maximum Likelihood (FIML) was used. The estimator used is WLSMV-Weighted Least Square Mean and Variance Adjusted. Standardised estimates (STDYX) reported in the article; all coefficients significant at *p* < 0.05 unless otherwise specified. Details of the full SEM output are in Supplementary Table A1.

5 To assess the ability of the SEM to reproduce the data, I referred to the CFI (Comparative Fit Index), RMSEA (Root Mean Square Error of Approximation) and SRMR (Standardized Root Mean Square Residual). The cut-off values are: (a) CFI: if the value is equal to 0.95, the model can be accepted, values above 0.90 are satisfactory; (b) RMSEA: values between 0 and 0.05 are considered indicators of a good fit; (c) SRMR, a value of less than 0.08 is generally considered a good fit.

6 To better interpret SEM model results, it is useful to clarify the distinction between total, direct, and indirect effects. The *total effect* represents the overall association between nativism and exclusionary labour market preferences, while the *direct effect* isolates the portion of this relationship that operates independently of other variables. The *indirect effects*, in turn, capture how this influence unfolds through mediators—here, perceived economic and cultural threats and intergroup distrust. Detailed output are in Supplementary Table A2.

7 The analysis employed a multi-group (cross-national) SEM using the *GROUPING = country* option. Following prior tests confirming configural and metric invariance but not scalar invariance, factor loadings for all latent constructs were constrained to equality across countries, while all structural paths were freely estimated (i.e., a metric-invariant model). Both the measurement and structural components were estimated simultaneously within a full SEM framework incorporating multiple mediating pathways. Model fit indices indicated an acceptable, though not optimal, fit (χ(539) = 7451.03, *p* < 0.001; RMSEA = 0.086, 90% CI [0.084, 0.088]; CFI = 0.878; TLI = 0.834). Given the model’s complexity and the multi-group design, these values fall within the range commonly regarded as adequate for cross-national comparative SEMs (Byrne, 2012). The largest contributions to the χ^2^ statistic originated from Germany and France, reflecting moderate cross-country heterogeneity without undermining the interpretability of the structural relations. Detailed model estimates, including fit indices and country-specific parameter tables, are available from the author upon request.

8 Tables presenting estimates of both the direct and indirect effects of Ethnic Majoritarianism and Civility on support for native employment priority in each country are provided in the Supplementary Table A3.
