# Supplementary material for: National identity as a lens on social inequality: a cross-national analysis of support for native employment priority
Source: Front Sociol. 2026 Feb 16;10:1657087. doi: 10.3389/fsoc.2025.1657087 (PMC12950609; doi:10.3389/fsoc.2025.1657087)
Supplement: Supplementary file 1 [file Table_1.docx]

Supplementary material

# Supplementary tables

# Table A1. Standardised estimates from the Full NITT SEM model based on EVS 2017 data across pooled dataset (Germany, France, Great Britain, Italy, Hungary, Poland, Portugal); n=12,183.

| **Path** | **Estimate** | **S.E.** | **z (Est./S.E.)** | **p-value** |
| --- | --- | --- | --- | --- |
| ETHNIC-MAJORITARIANISM BY ancestry | 0.850 | 0.005 | 170.655 | 0.000 |
| ETHNIC-MAJORITARIANISM BY born in country | 0.752 | 0.006 | 136.413 | 0.000 |
| ETHNIC-MAJORITARIANISM BY religion | 0.532 | 0.008 | 69.822 | 0.000 |
| CIVILITY BY [country] language | 0.592 | 0.009 | 67.937 | 0.000 |
| CIVILITY BY respect [country] laws/institutions | 0.377 | 0.01 | 38.127 | 0.000 |
| CIVILITY share [country] culture | 0.763 | 0.008 | 92.344 | 0.000 |
| DIFFERENCE IN FOREIGNERS BY other religion | 0.861 | 0.007 | 125.854 | 0.000 |
| DIFFERENCE IN FOREIGNERS BY other nationality | 0.956 | 0.007 | 133.821 | 0.000 |
| REALISTIC THREAT BY increase crime | 0.832 | 0.006 | 132.434 | 0.000 |
| REALISTIC THREAT BY burden welfare state | 0.819 | 0.006 | 129.969 | 0.000 |
| GLOBALISM BY close to Europe | 0.897 | 0.008 | 112.148 | 0.000 |
| GLOBALISM BY close to World | 0.700 | 0.008 | 91.261 | 0.000 |
|  |  |  |  |  |
| REALISTIC THREAT ON ETHNIC-MAJORITARIANISM | 0.288 | 0.016 | 18.562 | 0.000 |
| REALISTIC THREAT ON CIVILITY | 0.178 | 0.017 | 10.331 | 0.000 |
| REALISTIC THREAT ON GLOBALISM | -0.170 | 0.013 | -13.352 | 0.000 |
| REALISTIC THREAT ON COUNTRY ATTACHMENT | 0.022 | 0.012 | 1.816 | 0.069 |
| DISTRUST IN FOREIGNERS ON ETHNIC-MAJORITARIANISM | 0.285 | 0.015 | 18.556 | 0.000 |
| DISTRUST IN FOREIGNERS ON CIVILITY | -0.053 | 0.017 | -3.083 | 0.002 |
| DISTRUST IN FOREIGNERS ON GLOBALISM | -0.180 | 0.013 | -14.07 | 0.000 |
| DISTRUST IN FOREIGNERS ON COUNTRY ATTACHMENT | -0.013 | 0.012 | -1.114 | 0.265 |
| SYMBOLIC THREAT ON ETHNIC MAJORITARIANISM | -0.035 | 0.015 | -2.288 | 0.022 |
| SYMBOLIC THREAT ON CIVILITY | 0.238 | 0.016 | 14.487 | 0.000 |
| SYMBOLIC THREAT ON GLOBALISM | -0.099 | 0.012 | -8.108 | 0.000 |
| SYMBOLIC THREAT ON COUNTRY ATTACHMENT | 0.014 | 0.012 | 1.165 | 0.244 |
| NATIVE JOB PRIORITY ON GLOBALISM | -0.036 | 0.01 | -3.549 | 0.000 |
| NATIVE JOB PRIORITY ON ETHNIC MAJORITARIANISM | 0.326 | 0.013 | 25.322 | 0.000 |
| NATIVE JOB PRIORITY ON CIVILITY | 0.053 | 0.014 | 3.721 | 0.000 |
| NATIVE JOB PRIORITY ON REALISTIC THREAT | 0.292 | 0.01 | 28.773 | 0.000 |
| NATIVE JOB PRIORITY ON DISTRUST IN FOREIGNERS | 0.104 | 0.009 | 11.551 | 0.000 |
| NATIVE JOB PRIORITY ON COUNTRY ATTACHMENT | 0.004 | 0.009 | 0.48 | 0.632 |
| NATIVE JOB PRIORITY ON SYMBOLIC THREAT | 0.067 | 0.008 | 8.311 | 0.000 |
|  |  |  |  |  |
| COUNTRY ATTACHMENT WITH ETHNIC MAJORITARIANISM | 0.144 | 0.01 | 14.535 | 0.000 |
| COUNTRY ATTACHMENT WITH CIVILITY | 0.250 | 0.011 | 23.286 | 0.000 |
| COUNTRY ATTACHMENTWITH GLOBALISM | 0.481 | 0.008 | 59.285 | 0.000 |
| GLOBALISM WITH ETHNIC MAJORITARIANISM | -0.099 | 0.011 | -8.894 | 0.000 |
| GLOBALISM WITH CIVILITY | -0.025 | 0.012 | -2.062 | 0.039 |
| ETHNIC MAJORITARIANISM WITH CIVILITY | 0.614 | 0.009 | 65.848 | 0.000 |
| SYMBOLIC THREAT WITH DISTRUST | 0.082 | 0.01 | 8.006 | 0.000 |
| SYMBOLIC THREAT WITH REALISTIC THREAT | 0.176 | 0.011 | 16.41 | 0.000 |
| REALISTIC THREAT WITH DISTRUST | 0.244 | 0.011 | 21.739 | 0.000 |

# Table A2. Direct and indirect effects of ethnic-majoritarianism and civility on support for native employment priority. Standardised estimates from the Full NITT SEM model based on EVS 2017 data across pooled dataset (Germany, France, Great Britain, Italy, Hungary, Poland, Portugal), n=12,183.

| Path | Estimate | SE | P-value |
| --- | --- | --- | --- |
| Ethnic-majoritarianism (total) | 0.438 | 0.013 | 0.0 |
| Ethnic-majoritarianism (direct) | 0.326 | 0.013 | 0.0 |
| Indirect via symbolic/cultural threat | -0.002 | 0.001 | 0.03 |
| Indirect via intergroup distrust | 0.03 | 0.003 | 0.0 |
| Indirect via realistic/economic threat | 0.084 | 0.005 | 0.0 |
| Civility (total) | 0.116 | 0.015 | 0.0 |
| Civility (direct) | 0.053 | 0.014 | 0.0 |
| Indirect via symbolic/cultural threat | 0.016 | 0.002 | 0.0 |
| Indirect via intergroup distrust | -0.005 | 0.002 | 0.003 |
| Indirect via realistic/economic threat | 0.052 | 0.005 | 0.0 |

# Table A3. Direct and indirect effects of ethnic-majoritarianism and civility on support for native employment priority. Standardised estimates from the multi-group SEM model; EVS 2017 data – single country

**France** — SEM path estimates: direct and indirect effects of ethnic majoritarianism on support for native employment priority (standardised estimate), n=1,870

| Effect / path | Estimate (β) | SE | Z | P |
| --- | --- | --- | --- | --- |
| Total effect | 0.417 | 0.026 | 16.252 | 0.000 |
| Total indirect effect | 0.147 | 0.016 | 8.970 | 0.000 |
| Indirect via symbolic/cultural threat | −0.001 | 0.001 | −0.851 | 0.395 |
| Indirect via intergroup distrust | 0.034 | 0.008 | 4.341 | 0.000 |
| Indirect via realistic/economic threat | 0.114 | 0.015 | 7.460 | 0.000 |
| Direct effect | 0.271 | 0.028 | 9.540 | 0.000 |

**France** — SEM path estimates: direct and indirect effects of civility on support for native employment priority (standardised estimate), n=1,870

| Effect / path | Estimate (β) | SE | Z | P |
| --- | --- | --- | --- | --- |
| Total effect | 0.210 | 0.031 | 6.698 | 0.000 |
| Total indirect effect | 0.096 | 0.016 | 5.829 | 0.000 |
| Indirect via symbolic/cultural threat | 0.012 | 0.006 | 1.979 | 0.048 |
| Indirect via intergroup distrust | 0.001 | 0.004 | 0.159 | 0.874 |
| Indirect via realistic/economic threat | 0.084 | 0.015 | 5.751 | 0.000 |
| Direct effect | 0.114 | 0.032 | 3.519 | 0.000 |

**Germany** — SEM path estimates: direct and indirect effects of *ethnic -majoritarianism* on support for native employment priority (standardised estimate), n=2,167

| Effect / path | Estimate (β) | SE | Z | P |
| --- | --- | --- | --- | --- |
| Total effect | 0.239 | 0.060 | 3.991 | 0.000 |
| Total indirect effect | 0.016 | 0.037 | 0.420 | 0.674 |
| Indirect via symbolic/cultural threat | −0.009 | 0.007 | −1.240 | 0.215 |
| Indirect via intergroup distrust | 0.021 | 0.008 | 2.472 | 0.013 |
| Indirect via realistic/economic threat | 0.004 | 0.029 | 0.144 | 0.885 |
| Direct effect | 0.224 | 0.051 | 4.410 | 0.000 |

**Germany** — SEM path estimates: direct and indirect effects of *civility* on support for native employment priority (standardised estimate), n=2,167

| Effect / path | Estimate (β) | SE | Z | P |
| --- | --- | --- | --- | --- |
| Total effect | 0.228 | 0.078 | 2.933 | 0.003 |
| Total indirect effect | 0.218 | 0.053 | 4.123 | 0.000 |
| Indirect via symbolic/cultural threat | 0.037 | 0.016 | 2.354 | 0.019 |
| Indirect via intergroup distrust | 0.012 | 0.010 | 1.129 | 0.259 |
| Indirect via realistic/economic threat | 0.170 | 0.041 | 4.101 | 0.000 |
| Direct effect | 0.009 | 0.085 | 0.109 | 0.913 |

**Great Britain** — SEM path estimates: direct and indirect effects of *ethnic -majoritarianism* on support for native employment priority (standardised estimate), n=1,788

| Effect / path | Estimate (β) | SE | Z | P |
| --- | --- | --- | --- | --- |
| Total effect | 0.280 | 0.036 | 7.793 | 0.000 |
| Total indirect effect | 0.109 | 0.017 | 6.533 | 0.000 |
| Indirect via symbolic/cultural threat | 0.001 | 0.002 | 0.284 | 0.776 |
| Indirect via intergroup distrust | 0.009 | 0.005 | 1.946 | 0.052 |
| Indirect via realistic/economic threat | 0.100 | 0.016 | 6.244 | 0.000 |
| Direct effect | 0.171 | 0.036 | 4.695 | 0.000 |

**Great Britain** — SEM path estimates: direct and indirect effects of *civility* on support for native employment priority (standardised estimate), n=1,788

| Effect / path | Estimate (β) | SE | Z | P |
| --- | --- | --- | --- | --- |
| Total effect | 0.239 | 0.039 | 6.108 | 0.000 |
| Total indirect effect | 0.069 | 0.016 | 4.345 | 0.000 |
| Indirect via symbolic/cultural threat | 0.015 | 0.006 | 2.523 | 0.012 |
| Indirect via intergroup distrust | −0.005 | 0.004 | −1.525 | 0.127 |
| Indirect via realistic/economic threat | 0.059 | 0.014 | 4.370 | 0.000 |
| Direct effect | 0.170 | 0.040 | 4.302 | 0.000 |

**Hungary** - SEM path estimates: direct and indirect effects of *ethnic majoritarianism* on support for native employment priority (standardised estimate), n=1,514

| Effect/path | Estimate. Β | SE | Z | P |
| --- | --- | --- | --- | --- |
| Total effect - ethnic majoritarianism | 0.142 | 0.041 | 3.464 | 0.001 |
| Total indirect | 0.112 | 0.020 | 5.477 | 0.000 |
| Indirect via symbolic/cultural threat | −0.004 | 0.003 | −1.342 | 0.180 |
| Indirect via intergroup distrust | 0.013 | 0.010 | 1.343 | 0.179 |
| Indirect via realistic/economic threat | 0.103 | 0.017 | 5.911 | 0.000 |
| Direct effect | 0.030 | 0.044 | 0.689 | 0.491 |

**Hungary** - SEM path estimates: direct and indirect effects of *civility* on support for native employment priority (standardised estimate), n=1,514

| Effect / path | Std. Effect (β) | SE | Z | P |
| --- | --- | --- | --- | --- |
| Total effect | 0.169 | 0.042 | 3.980 | 0.000 |
| Total indirect | 0.039 | 0.016 | 2.432 | 0.015 |
| Indirect via symbolic/cultural threat | 0.009 | 0.006 | 1.670 | 0.095 |
| Indirect via intergroup distrust | −0.004 | 0.003 | −1.085 | 0.278 |
| Indirect via realistic/economic threat | 0.033 | 0.014 | 2.361 | 0.018 |
| Direct effect | 0.130 | 0.042 | 3.110 | 0.002 |

**Italy** - SEM path estimates: direct and indirect effects of *ethnic majoritarianisms* on support for native employment priority (standardised estimate), n=2,277

| Effect / path | Estimate (β) | SE | Z | P |
| --- | --- | --- | --- | --- |
| Total effect | 0.375 | 0.027 | 13.955 | 0.000 |
| Total indirect | 0.138 | 0.015 | 9.205 | 0.000 |
| Indirect via symbolic/cultural threat | 0.007 | 0.003 | 2.474 | 0.013 |
| Indirect via intergroup distrust | 0.027 | 0.006 | 4.655 | 0.000 |
| Indirect via realistic/economic threat | 0.104 | 0.013 | 7.911 | 0.000 |
| Direct effect | 0.237 | 0.026 | 9.001 | 0.000 |

**Italy** - SEM path estimates: direct and indirect effects of *civility* on support for native employment priority (standardised estimate), n=2,277

| Effect / path | Estimate (β) | SE | Z | P |
| --- | --- | --- | --- | --- |
| Total effect | 0.019 | 0.028 | 0.655 | 0.512 |
| Total indirect effect | 0.044 | 0.015 | 2.984 | 0.003 |
| Indirect via symbolic/cultural threat | 0.008 | 0.003 | 2.611 | 0.009 |
| Indirect via intergroup distrust | −0.004 | 0.005 | −0.826 | 0.409 |
| Indirect via realistic/economic threat | 0.040 | 0.012 | 3.226 | 0.001 |
| Direct effect | −0.026 | 0.026 | −0.993 | 0.321 |

**Poland**- SEM path estimates: direct and indirect effects of *ethnic majoritarianisms* on support for native employment priority (standardised estimate), n=1,352

| Effect / path | Estimate (β) | SE | Z | P |
| --- | --- | --- | --- | --- |
| Total effect | 0.447 | 0.070 | 6.345 | 0.000 |
| Total indirect effect | 0.167 | 0.033 | 5.129 | 0.000 |
| Indirect via symbolic/cultural threat | −0.003 | 0.004 | −0.638 | 0.523 |
| Indirect via intergroup distrust | 0.057 | 0.016 | 3.545 | 0.000 |
| Indirect via realistic/economic threat | 0.113 | 0.026 | 4.268 | 0.000 |
| Direct effect | 0.280 | 0.067 | 4.149 | 0.000 |

**Poland**- SEM path estimates: direct and indirect effects of *civility* on support for native employment priority (standardised estimate), n=1,352

| Effect / path | Estimate (β) | SE | Z | P |
| --- | --- | --- | --- | --- |
| Total effect | −0.043 | 0.072 | −0.598 | 0.550 |
| Total indirect effect | −0.041 | 0.032 | −1.280 | 0.201 |
| Indirect via symbolic/cultural threat | 0.002 | 0.003 | 0.624 | 0.533 |
| Indirect via intergroup distrust | −0.015 | 0.014 | −1.075 | 0.282 |
| Indirect via realistic/economic threat | −0.029 | 0.025 | −1.129 | 0.259 |
| Direct effect | −0.002 | 0.066 | −0.034 | 0.973 |

**Portugal** - SEM path estimates: direct and indirect effects of *ethnic majoritarianisms* on support for native employment priority (standardised estimates), n=1,215

| Effect / path | Estimate (β) | SE | Z | P |
| --- | --- | --- | --- | --- |
| Total effect | 0.252 | 0.064 | 3.939 | 0.000 |
| Total indirect effect | 0.109 | 0.028 | 3.831 | 0.000 |
| Indirect via symbolic/cultural threat | −0.003 | 0.006 | −0.465 | 0.642 |
| Indirect via intergroup distrust | −0.006 | 0.012 | −0.475 | 0.635 |
| Indirect via realistic/economic threat | 0.117 | 0.024 | 4.823 | 0.000 |
| Direct effect | 0.143 | 0.064 | 2.221 | 0.026 |

**Portugal**- SEM path estimates: direct and indirect effects of *civility* on support for native employment priority (standardised estimate), n=1,215

| Effect / path | Estimate (β) | SE | Z | P |
| --- | --- | --- | --- | --- |
| Total effect | 0.067 | 0.065 | 1.028 | 0.304 |
| Total indirect effect | 0.008 | 0.026 | 0.300 | 0.764 |
| Indirect via symbolic/cultural threat | 0.003 | 0.006 | 0.439 | 0.661 |
| Indirect via intergroup distrust | 0.026 | 0.013 | 2.076 | 0.038 |
| Indirect via realistic/economic threat | −0.021 | 0.020 | −1.067 | 0.286 |
| Direct effect | 0.059 | 0.063 | 0.950 | 0.342 |

**
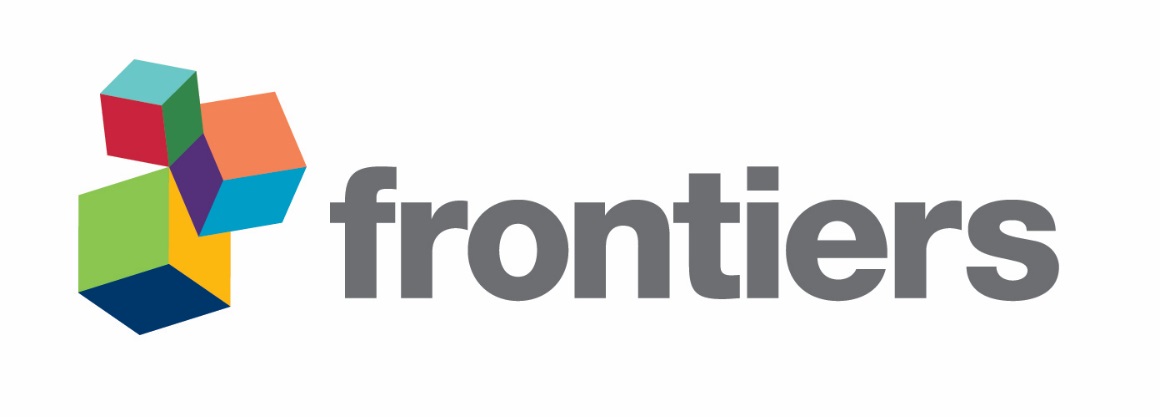
**
